# Transparent to Black Electrochromism—The “Holy Grail” of Organic Optoelectronics

**DOI:** 10.3390/polym11020273

**Published:** 2019-02-06

**Authors:** Tomasz Jarosz, Karolina Gebka, Agnieszka Stolarczyk, Wojciech Domagala

**Affiliations:** 1 Department of Physical Chemistry and Technology of Polymers, Silesian University of Technology, 9 Strzody Street, 44-100 Gliwice, Poland; Karolina.Gebka@polsl.pl (K.G.); Agnieszka.Stolarczyk@polsl.pl (A.S.); 2 Department of Inorganic Chemistry, Analytical Chemistry and Electrochemistry, Silesian University of Technology, 6 Krzywoustego Street, 44-100 Gliwice, Poland; Tomasz.Jarosz@polsl.pl

**Keywords:** electrochromism, electrochromic device, displays, smart window, e-paper, pi-conjugated molecule, redox doping, electrochemistry, polymer

## Abstract

In the rapidly developing field of conjugated polymer science, the attribute of electrochromism these materials exhibit provides for a multitude of innovative application opportunities. Featuring low electric potential driven colour change, complemented by favourable mechanical and processing properties, an array of non-emissive electrochromic device (ECD) applications lays open ahead of them. Building up from the simplest two-colour cell, multielectrochromic arrangements are being devised, taking advantage of new electrochromic materials emerging at a fast pace. The ultimate device goal encompasses full control over the intensity and spectrum of passing light, including the two extremes of complete and null transmittance. With numerous electrochromic device architectures being explored and their operating parameters constantly ameliorated to pursue this target, a summary and overview of developments in the field is presented. Discussing the attributes of reported electrochromic systems, key research points and challenges are identified, providing an outlook for this exciting topic of polymer material science.

## 1. Concepts and Considerations

Electrochromism and electrochromic devices have always been considered a field of significant research interest. Transmissive to black electrochromism, involving two most sterling of colourations, has long stood out as the ultimate exploit of this redox driven phenomenon, holding promise for developing low-cost, low energy demand passive display, or light modulation technology. References to this topic can be found in a number of reviews [1,2,3,4], but no broader address of the subject has yet been made. With the advent of commercial demand for such systems, one example being the e-paper technology, the subject is garnering the dedicated attention of material scientists, making it prudent to summarise existing developments and provide organisational structure for future research developments.

### 1.1. What Is Black Electrochromism?

Apart from general considerations for electrochromism, black electrochromism is missing clear and inclusive delineation. The only definition is being hinted at by Liou et al., as “*an extensive absorption ranging of 400–750 nm required for a black electrochromism*” [5]. Although this definition describes the dark state adequately, it overlooks the crucial complementary bleached state. This necessitates a more comprehensive criterion, in order to contain and differentiate among the electrochromes applicable for transmissive to black electrochromic devices. Therefore, we postulate the following description of a black to transmissive electrochromic device: *A device capable of reversibly, electrochemically switching between at least one dark and one bleached state, exhibiting uniform, significant absorption, covering the 400–750 nm range in the dark state and either partial, low-absorbance coverage or no absorption coverage in the bleached state.*

### 1.2. Black Electrochromism—Potential and Applications

Typical electrochromic devices act selectively, altering light, passing through them, by filtering out individual wavelength ranges. In contrast, black electrochromic devices grant control over the visible light intensity, indiscriminately of wavelength. This feature predestines them to have significant use in smart windows, enabling a decrease of expenses incurred when air conditioning and cooling illuminated areas [4,6,7]. The same principle applies to “smart mirrors” as well, as exemplified by the commercial success of car rear view mirrors manufactured by Gentex [8]. Furthermore, high resolution devices of this type are also in demand for thin flexible e-paper displays as they allow some similarity to classic paper.

### 1.3. Potential Black Electrochromic Materials

Theoretically, electrochromism may occur in any class of chemical compounds. Although it is important to consider novel materials, extensive literature is available, detailing a range of promising electrochrome types. Those include: transition metal oxides, prussian blue systems, viologens, conjugated polymers, transition metal complexes and metal phthalocyanines [9,10]. Of those, transition metal oxides, viologens and conjugated polymers have been reported to exhibit bleached and dark coloured states [6,11,12]. Each of those systems has its strong and weak points, therefore, each may excel when presented with different requirements. However, due to the multitude possible conditions when considering application of a system, versatility is of prime importance. This leads to considering potential electrochromic materials based not only on their spectroelectrochemical characteristics, but also on their “secondary” properties, which may validate application in a given environment.

The implementation of conducting polymers as active materials in black electrochromic devices is found to be beneficial due to a number of features intrinsic to conjugated polymers. These are advantageous, concerning both the final operational parameters of hypothetical devices, as well as the basic processes leading to fabrication of such devices. The chemical structure of polymer electrochromes comprises a hydrocarbon skeleton, often supplemented with non-metal and occasionally with metalloid heteroatoms. Therefore, they are viewed as relatively inexpensive, environmentally benign and possibly fall into the sustainable technology category. Inorganic systems, on the other hand, utilise transition metals, leading to increased cost and environmental issues. This can be illustrated by a silver based device, developed by Kobayashi et al. [13]. Although it exhibits stupendous spectroelectrochemical properties, any commercial scale application would have to factor in the volatile price of silver in device unit cost breakdown, putting pressure on the economic viability of this technology.

Conjugated polymers benefit from the possibility of fine tuning their properties, through structural modification. Therefore, transmissive to black electrochromic systems may be obtained not only through discovery, but also through design of new systems, as well as modification of existing ones [5,10,14,15]. Apart from spectroelectrochemical characteristics, the solubility of the polymer may be significantly altered via structural modification [10]. This leads to the applicability of solution-based processing methods, which are particularly adaptable and energy efficient. Furthermore, if a sufficiently soluble polymer may not be obtained easily, electrochemical polymerisation may be employed, allowing for deposition of a conductive polymer film from a solution of the appropriate monomer. This method also allows for adaptation to complex shapes of the substrate to be coated, as well as significant control of the thickness and oxidation state of the coating itself.

### 1.4. Approaches to Black Electrochromism in Conjugated Polymers

Starting from the postulated definition, a series of idealised models may be derived. The simplest of these assumes the existence of a black state and a colourless state. The former would feature a single, absorption peak, spanning the whole visible spectrum, while the latter exhibits no absorption in this range. In this scenario, the electric stimulus would serve to modify the molecule, so as to induce a reversible transition to a species exhibiting no absorption in the visible range. This reasoning can be transferred onto the grounds of band theory in conducting polymers and be thought of as the results of an electrochromic species undergoing doping/dedoping processes. Furthermore, the possibility of p-doping and n-doping, even if often limited to only one doping type, allows us to consider the presence of an additional coloured state, when proceeding from the absorbing “dark” state and the modified, colourless “bleached” state. The simplicity of such a system would be an important advantage when discussing the operating parameters of a hypothetical electrochromic device, however, non-uniform absorption may result in a tinted dark state.

**Figure 1:**
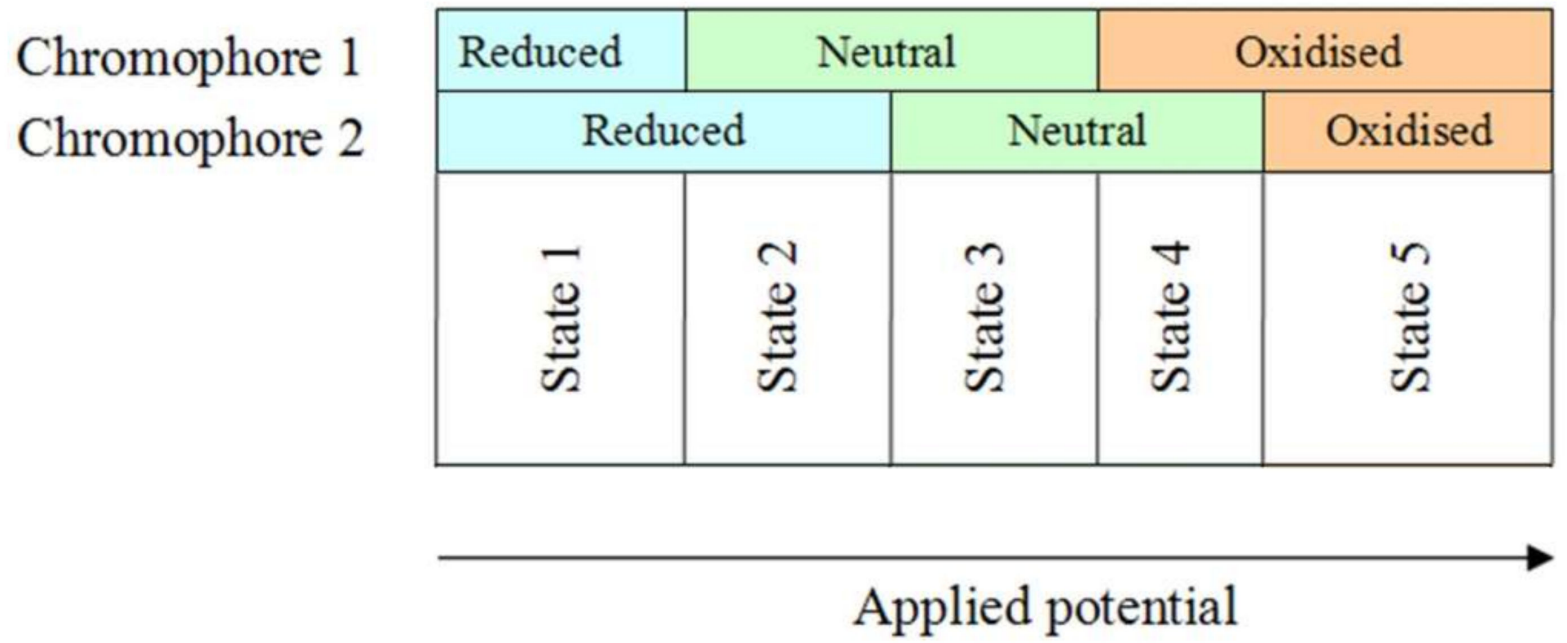
Theoretically attainable chromic states for a single working electrode modified with two polymer electrochromes.

Elaborating on this approach, a more sophisticated model can be derived, in which the superposing absorption bands originate from different, electroactive chromophores. This may be achieved by employing a more complex molecular structure or by using a mixture of electrochromic species. The presence of multiple chromophores leads to a more uniform absorption in the visible range. Furthermore, should the redox potentials of the chromophores be non-identical, it would theoretically be possible to produce stable transition states, characterised by individual chromophores existing in different oxidation states (Figure 1). The now-famous “Butterfly-ECD” is an excellent example of this type of configuration, utilising a mixture of two electrochromic polymers solution cast on a common working electrode [16].

The number of individual coloured states that could be achieved is determined by the individual electronic properties of each chromophore. It is worth noting that a single material can contain numerous types of chromophores and be able to achieve multielectrochromism by itself. Even so, in the case of conjugated polymers, the chromophores tend to interact with each other through the conjugated bond system, exchanging charges and often presenting issues with the stability of the intermediate coloured states. Nevertheless, the ability to work with multiple electroactive chromophores can give an extremely broad range of coloured states, possibly even enabling the development of transmissive to black devices showing gradual darkening that could be held at any particular level between fully transmissive and black. The additional flexibility of optical properties is not without consequences, however, as constraints related to the spectroelectrochemical properties of each constituent electrochrome need to be considered, as well as their impact on the resultant device parameters.

Theoretically, it is not necessary for the individual absorption bands to originate from the same conjugated polymer layer, even should practical considerations favour simplification of the system. Due to the additive character of light absorption, different wavelengths can be subtracted from the spectrum sequentially. Upon passing through all the layers, or interacting with all the chromophores, (almost) no visible light persists, thus yielding a black coloured state. This approach allows us to utilise a number of electrochromes exhibiting limited absorption, in order to obtain absorptive coverage of the entire visible spectrum. The prime drawback of this approach, however, is that the above principle applies to the bleached state as well. Therefore, even negligible absorption of each individual electrochrome in its respective bleached state, may result in a discernible net absorption, limiting optical contrasts attainable in hypothetical devices. Even despite this, the approach remains feasible if materials, exhibiting sufficiently low bleached state absorption, can be found.

**Figure 2:**
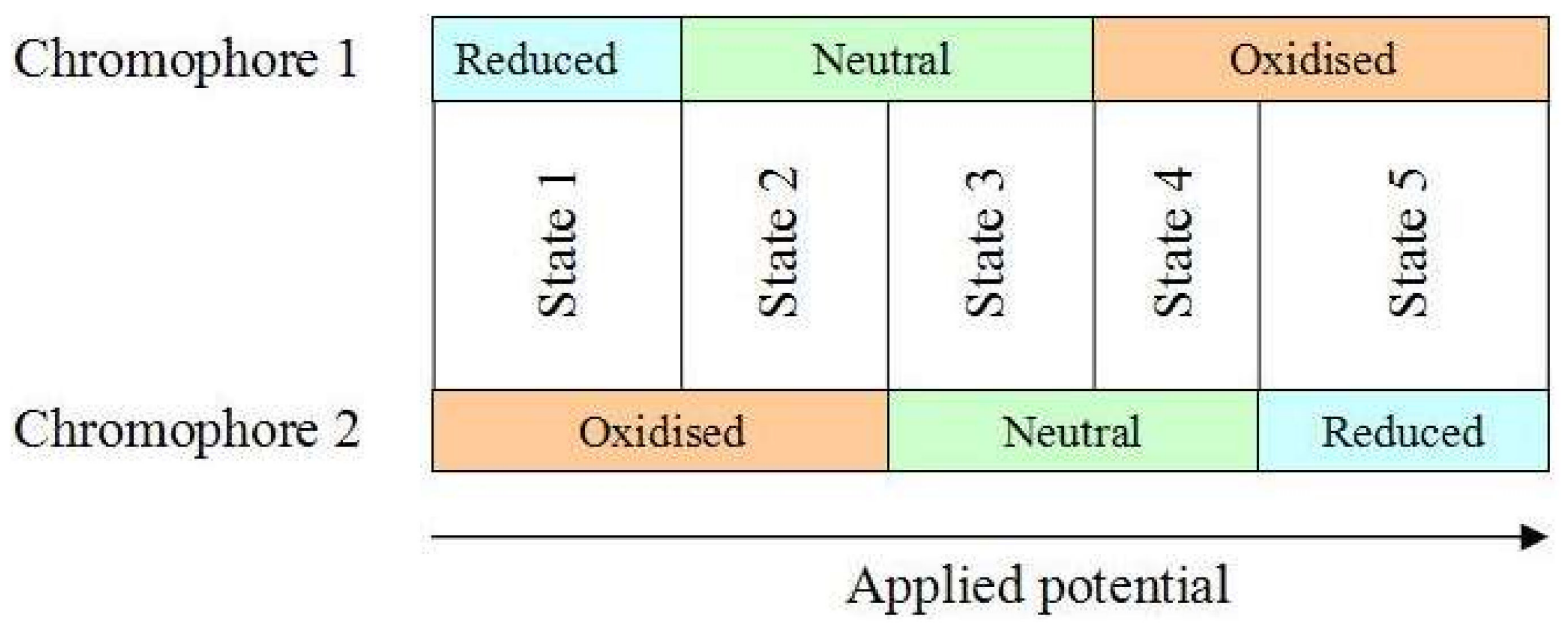
Theoretically attainable chromic states for a two-electrode system, where each electrode has been modified with a different polymer electrochrome.

The existence of separate electrochromic layers enables the simultaneous use of both the working and counter electrodes of a device. The utilisation of electrochromes coated onto the working and counter electrodes introduces the concept of electrochromic complementarity. This is realised through a pair of electrochromes, one of which is bleached in its oxidised state (anodically bleaching) and the other is bleached in its reduced state (cathodically bleaching). One is coated onto the working electrode and the other onto the counter electrode. Upon oxidation of one of the pair, the other undergoes reduction (Figure 2) and vice versa. The practical application of this architecture is exemplified by the electrochromic device reported by Liang et al. [17], which utilises both anodically- and cathodically-colouring systems.

Effectively, this yields a device switching between both polymers in the bleached state and in the coloured state. Thus, the choice of applicable electrochromic materials is significantly widened. The advantage of this design is the mutual accommodation of doping charge of both electrochrome layers, in that when anodically colouring electrochrome undergoes p-doping (oxidation), the electrons withdrawn from it are taken up (through the powering circuit) by the cathodically colouring electrochrome undergoing n-doping. Likewise, charge segregation of electrolyte ions is facilitated by complementary uptake of anions by the p-doped electrochrome, and cations by the n-doped electrochrome, boosting the colouration efficiency of the cell.

Summarising, each of the approaches discussed above is equally valid and scientifically valuable and the pursuit of each presents its unique set of challenges and advantages. When progressing from simpler models towards the sophisticated ones, constraints related to the spectral parameters of the electrochrome are being relaxed. This, however, happens at the cost of constraints related to the operating parameters of the device progressively becoming stricter.

### 1.5. Material Considerations for Electrochromic Devices

According to the Beer–Lambert–Bouguer law, light absorption is correlated with the molar absorption coefficient (ε), optical path length (l) and the number density of absorbers in the absorbing layer (N). Optical contrast arises from the difference in the values of ε(λ) in the bleached and dark states, and, for a given material, is an intrinsic parameter. The number density of absorbers is related to the density and morphology of a material, which in many cases are difficult to adjust. Upon oxidation or reduction of the polymer electrochrome, chromophore centres may be either activated or deactivated, making their number density directly dependent on the redox state of the electrochromic material. When processing a given material, its absorption is typically controlled through polymer layer thickness. This leaves potential for optimisation, as film thickness also affects the electrochemical and electrical properties of the polymer layers.

Theory focuses on the fact that the active polymer layers within an electrochromic device feature optical absorption. Therefore, the absorbance and opacity of electrodes, supporting electrolyte and other components of the device are often disregarded, being taken as transparent in the visible range. Such electrolytes are known, although non-absorbing systems may still exhibit some degree of light scattering, contributing to the opacity of the electrochromic assembly. Electrodes are a different case, however. Conductivity of crystalline metalloid-based semiconductors is a function of their band gap energy, and common band gap values of those materials afford them discrete or diffuse absorption features in the Ultra Violet (UV), Visible (Vis), or Near Infra-Red (NIR) regions. The importance of the above is that, if the transmittance of the abovementioned “passive” components of a device is less than 100%, it will limit the optical contrast of a given device, independent of the electrochrome.

Indium-tin oxide (ITO) coatings, deposited on substrates such as poly(ethylene terephthalate) (PET), glass and quartz are by far the most common type of “optically transparent electrodes”. Those ITO films feature high transmittance, averaging at about 80% across the visible spectrum, although the two transition metals are unfavourable from an environmental point of view. Kuwana and Heineman [18] discussed the characteristics of a variety of electrodes, both conducting deposits on optically transparent substrates and metal wire meshes. Further works were reported, concerning carbon-based electrodes [19,20]. The transmittance exhibited by ITO electrodes, however, remained unrivalled until the recent development of graphene-based systems [21].

### 1.6. Commonly Encountered Architectures of Electrochromic Devices

**Figure 3:**
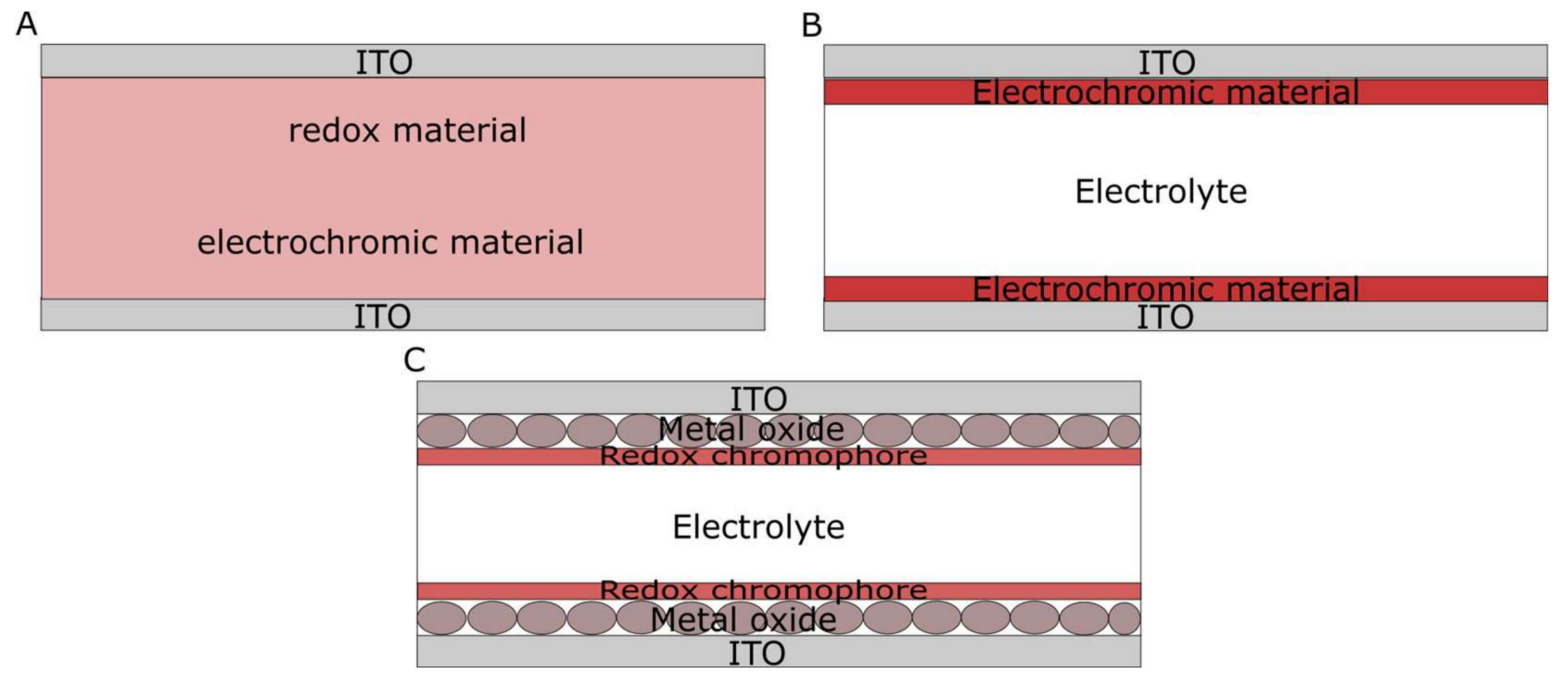
Schemes of three different designs of electrochromic devices: (**A**) First type with polymer matrix; (**B**) second type with thin polymer films applied on ITO; (**C**) third type with thin film of metal oxide modified by a redox chromophore.

In terms of their design, electrochromic devices (ECDs) can be divided into three main types (Figure 3) [22].

The first type, self-bleaching ECD, contains an electrolytic matrix with dispersed electrochromic molecules and redox agents between two optically transparent electrodes (OTEs), most commonly glass plates coated with the conducting indium-tin oxide (ITO). In order to maintain its coloured state, this type of device requires voltage to be constantly applied, as otherwise redox agents would restore the open circuit equilibrium colouration of the electrochromic material [22].

The second type, thin film based ECD, consists of two electrochromic material layers (each coated onto an OTE), respectively colouring upon their oxidation (anodically) and reduction (cathodically), with a polyelectrolyte layer sandwiched between them, acting as an interface and as a reservoir of charge compensating ions [23]. Concurrent complementary doping of both electrochromic layers minimises build-up of uncompensated charges at the electrode–electrolyte interface, diminishing undesired polarisation resistance of the electrochromic cell, and forestalling detrimental electrolysis of the electrolyte salt.

The third type, metal oxide based ECD, consists of two glass electrodes covered by thin, mesoporous metal oxide films with electrochromic compounds attached to them. This type of ECD generally shows higher colour switching rates than those typically reported for the first type ECD. The improved dynamics are due to the polycrystalline, porous metal oxide films; the individual crystallites comprising the films are very fine, having a high specific surface area. The large surface area of the electrodes greatly facilitates ion diffusion into the electrochromic material layers, avoiding the drawbacks of monolithic layer ECDs [24]. A prime example of such type of device is reported by Cummins et al., utilising anodically- and cathodically-colouring electrochromes. The colouring/bleaching times of the reported ECD are on the order of 200–600 ms, showcasing the potential of this device architecture [25].

Apart from the mainstream designs, numerous minor modifications have been reported in the bid to improve the properties of ECDs.

One such modification, compatible with each of the three ECD types, is to change the structure of ITO from flat to more porous by drop-casting dispersed nanoparticles of ITO onto planar ITO. Devices comprising a planar ITO working electrode and a modified (as mentioned above) ITO counter-electrode were reported. The electrodes were coated with cathodically- and anodically-colouring polymers respectively. The advantage of this device is in improved control over the individual coloured states, attributed by the authors to the increased double layer capacitance of the surface modified ITO–electrolyte solution interface [17].

The use of silver nanoparticles has also been reported in third type ECDs, yielding favourable electrochromic properties. In the work of Jeong et al., Ag nanoparticles were used to obtain a completely black state in an ECD using 3D-nanostructured ITO. ECDs with high stability were prepared, which could also attain a mirror state and a transparent state. Average transmittance values of T_avg_ > 73.76% in the transparent state, and reflectance value of R_avg_ > 79.77% in the mirror state, and R_avg_ > 8.78% for black state were reported respectively in the wavelength range from 400 to 700 nm [26].

**Figure 4:**
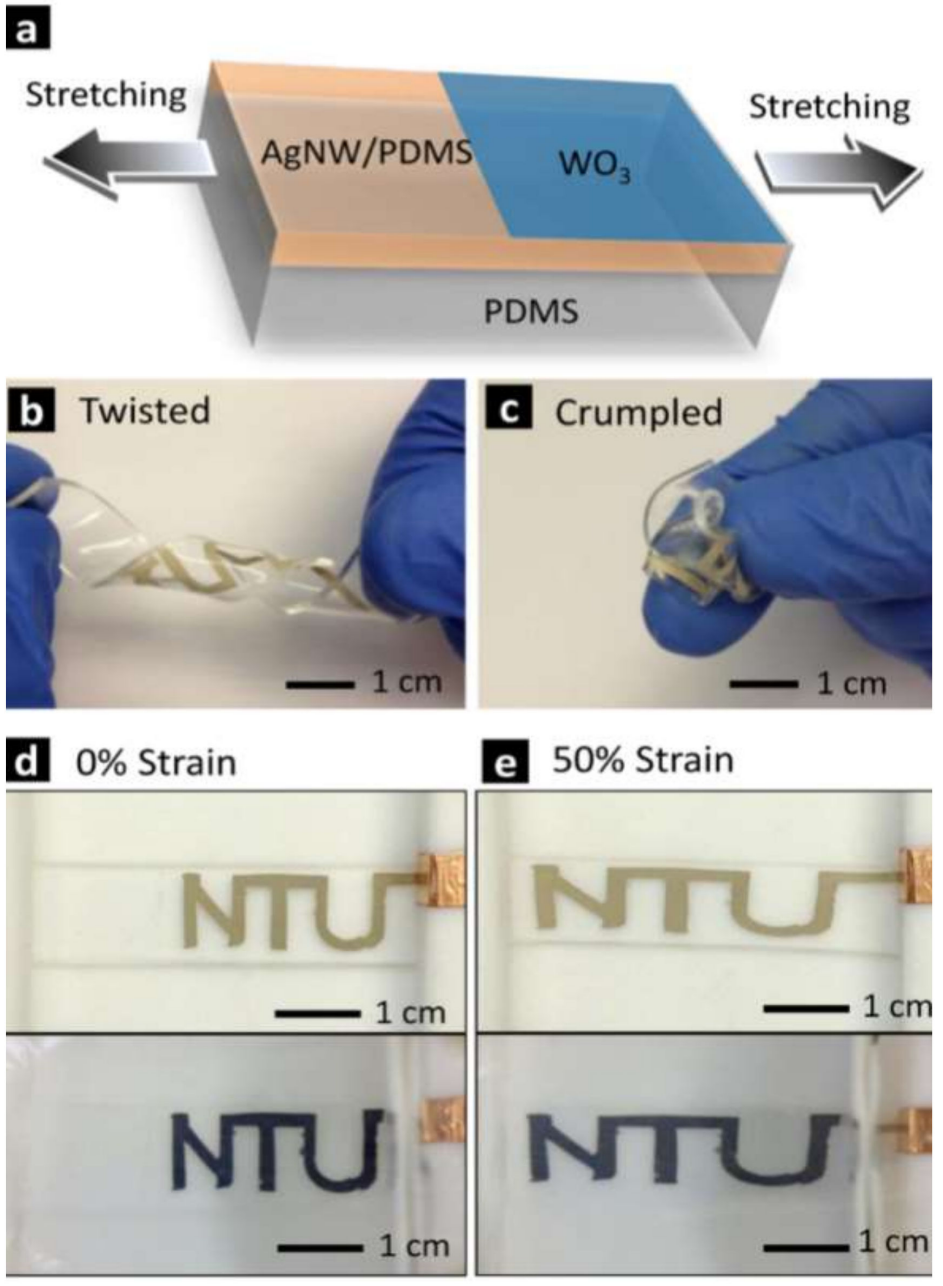
Flexible and stretchable electrochromic devices (ECDs) presented by Yan at al. Reprinted with permission from [27]. Copyright 2014, American Chemical Society.

To obtain flexible and stretchable ECDs (Figure 4), Yan et al. introduced an Ag nanowire electrode, consisting of Ag nanowires coated onto a polydimethylsiloxane susbtrate. WO_3_ electrodes were deposited onto the Ag nanowires, resulting in a device showing good optical contrasts and colour switching times, while appearing to be largely unaffected by various mechanical operations (folding, crumpling) [27]. Cai et al. reported a similar device, but managed to suppress oxidation of metal based electrode by coating the nanowire film with a thin layer of poly(3,4-ethylenedioxythiophene)—polystyrene-sulfonate (PEDOT:PSS) [28].

## 2. Pursuing the Goal

**Figure 5:**
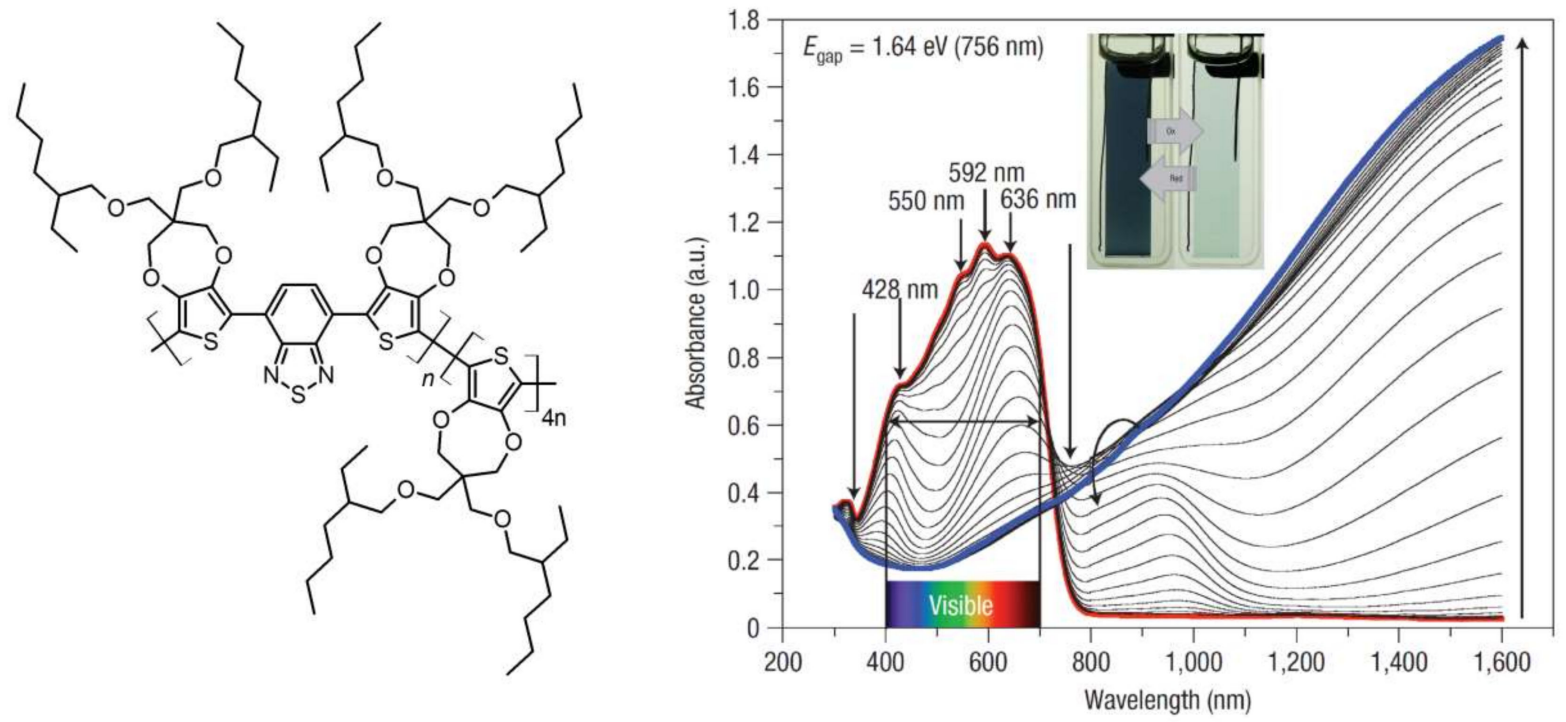
Spectroelectrochemistry of a copolymer of alkoxy derivatised poly(3,4-propylenedioxythiophene)-ProDOT and 2,1,3-benzothiadiazole, demonstrating black to transmissive switch upon doping of the polymer layer deposited at ITO electrode. Reprinted with permission and adapted from [30]. Copyright 2008, Springer Nature.

Pozo-Gonzalo first reported an electrochromic polymer with a truly spectroscopic black electrochromic state [29], however, it was not until the works of Reynolds et al. [30,31], that the concept of a transmissive to black colour switching was introduced (Figure 5). Therefore, even though prior works could have touched upon materials falling under the postulated definition, transmissive and/or black colour traits of polymer electrochromes reported therein could have gone underexposed due to lack of uniform nomenclature of the topic.

One of the first systems, exhibiting, what was later dubbed, transmissive-to-black electrochromism, was reported by Welsh et al. [32] for devices based on poly(3,4-propylenedioxythiophene) and its derivative. Although neither system showed a truly transparent transmissive state or a “true black” state, the low transmittance of the reduced polymers was sufficient to produce a tinted black appearance. Even though these were the first such devices, an impressive maximum optical contrast value of even 78% is reported for 578 nm. Similarly, although the stability of the functional parameters of the device is evaluated only for a very short time span, no discernible optical contrast losses are observed and colouration efficiency of the devices is reported to be on the order of 200 cm^2^/C.

Somani and Radhakrishnan [33] were one of the first to review the various electrochromic systems, both organic and inorganic in nature. Interestingly, many of the classes of compounds mentioned have later been used to produce transmissive-to-black devices (e.g., viologen derivatives). It is also worth noting that, from among the mentioned transition metal compounds, iridium(III) hydroxide can, by itself, be considered an early example of such a transmissive-to-black electrochromic material. Looking back on the authors’ postulates from the perspective of more than 15 years of research into all-polymer electrochromic devices, the kinetics of their operation, colour switching speeds in particular, predispose them to static light filtering (electrochromic windows) or display (adaptable signage) technologies.

Another pioneering review of electrochromic materials was compiled by Rowley and Mortimer [9], detailing most of the classes of compounds discussed by Somani, but also including systems such as metal phthalocyanines and metallopolymers.

In their review of electrochromic materials, Argun et al. [34] focused solely on electrochromic conjugated polymers. The authors indicate the methods most commonly used to investigate the properties of electrochromic polymers: spectroelectrochemistry, colorimetric and reflectance analyses, as well as colouration switching experiments. The different types of electrochromic devices (absorptive/transmissive, reflective and patterned) are also briefly discussed, with the authors seeing patterned ECDs as solutions for display technologies rather than as “smart” windows.

**Figure 6:**
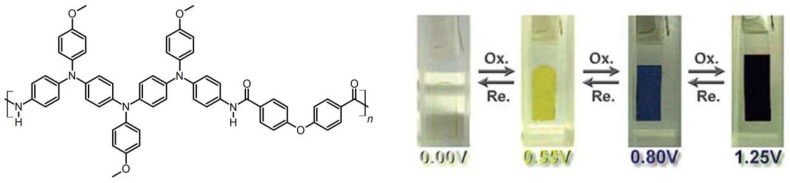
Polyamide composed of methoxy functionalised oligotriarylamine and diphenyl ether carboxylic acid units, demonstrating spectacular basic colour multielectrochromism, involving neat transmissive and black states. Reprinted with permission and adapted from [5]. Copyright 2012, The Royal Society of Chemistry.

The development of transmissive-to-black electochromic device [35] (Figure 6) is reported in detail by Yen and Liou [5] in the form of a review, encompassing all the work being done on electrochromic triarylamine-based materials capable of this remarkable feat. Where the initial works were focused on electrochromic polyamides, the attention of the Authors soon shifted towards poly(amide-imides) and, later, towards extending the triphenylamine system. The latter proved to be a fruitful modification to some extent, as while the electrochromic performance of the materials could be increased by extending the triarylamine core, further extensions eventually lead to the collapse of the desired colour traits (impure black coloured state, opaque, or tinted bleached state).

Another approach, which can be applied to produce both multi-coloured and transmissive-to-black electrochromic devices, is the use of more than one electrochromic material. In this case, Vasilyeva et al. [16] compared the performance of a “classical” electrochromic device, based on a donor-acceptor copolymer, with a “dual active layer” device, based on homopolymers of the two co-monomers of the aforementioned copolymer. Interestingly, while the “dual” devices show optical contrast values comparable to or even slightly higher than the “classical” devices, their colour switching times are significantly longer, and their coloration efficiency values are also significantly lower. The longer colour switching times for the “dual” devices are thought by the authors to stem from an inhomogeneous interface between the two polymers, which hinder ion and charge transport, highlighting the role of interfacial charge transfer processes in the overall performance of an electrochromic device.

This approach has recently been furthered by Savagian et al. [36], who report three polymer blends, exhibiting transmissive-to-black colour switching. Devices produced using these blends are compared with counterparts based on ECP-Black and although they achieve similar optical contrast values (on the order of 40%), noticeably lower coloration efficiency values are seen (approx. 303 cm^2^/C in the case of ECP-black and approx. 243 cm^2^/C for the reported CMY blend). A paramount advantage of the colour formulation strategy presented therein, though, is the smooth transition of the electrochromic polymer blend through intermediate shades of grey upon application of a potential bias ramp. This feature of uniform attenuation grading across the full visible light range constitutes an important advance towards developing organic based achromatic light modulation technology, suitable for tint-free smart windows or low-cost flexible passive greyscale displays.

Amb et al. [2] have revisited the subject of electrochromism with a review on the approaches to attaining various colourations of solution-processable electrochromic materials. This includes discussion of both cathodically and anodically colouring polymers, as well as brief overviews of the most remarkable systems for achieving both black colourations and other coloured states. The authors mention some works aimed at achieving black electrochromism, mostly focusing on the use of polymers and copolymers of benzothiadiazole and 3,4-alkylenedioxythiophenes. Both film and device characterisation methods are discussed, along with a number of solution processing methods for preparing polymer layers.

Where many authors design and synthesise well-defined, perfectly tailored copolymers to serve as transmissive-to-black electrochromic materials, Öktem et al. [37] overturn this viewpoint, by showing that such electrochromism can also be achieved by a highly statistic copolymer. Although benzotriazole and thiophene are the main repeat units constituting the authors’ copolymers, another type of repeat units is also interspersed within the copolymer chains. Three different “additional” repeat units have been used in this way, yielding three different copolymers with similar highest occupied molecular orbital (HOMO) and lowest unoccupied molecular orbital (LUMO) energies, due to the relative similarity of the repeat units. Interestingly, while all three copolymers show significant, albeit non-uniform absorption in the Vis range at open circuit potential, bleaching takes place upon their oxidation, resulting in transmissive grey states. Although relatively low optical contrasts (up to 23% at 600 nm) are reported, colour switching is relatively rapid, with the shortest switching time being reported as 0.7 s.

Buasri et al. [38] report an interesting approach to the fabrication of an electrochromic device, formulating the device with a liquid pyrrole monomer and electrolyte layer sandwiched between two graphene-coated recycled plastic films. When potential is applied to the liquid layer, the monomer undergoes electrochemical polymerisation and, quite remarkably, produces a film thick enough to show electrochromism. Although the authors did not report on the details of the operating parameters achieved for this device, it is worth noting that, apart from the extreme yellow and black coloured states, an intermediate colourless state was also observed.

Although polycyclic aromatic hydrocarbons (PAHs) have received much publicity as carcinogenic air contaminants, their large conjugated bond systems are the source of some favourable properties, such as very efficient fluorescence and, as found recently, collective optical excitations. Interestingly, they can also be utilised as active layers in electrochromic devices, as shown by Stec et al. [39]. PAHs typically show absorption in the UV range in their uncharged state, developing Vis-range absorption only upon either p- or n-doping. As such, while the transmissive state of a hypothetical transmissive-to-black device could easily be achieved, its coloured state requires a combination of PAHs to achieve uniform absorption in the Vis range, yielding a “true black” state. In this case, benzo[a]pyrene and anthracene anions were sufficient to yield such an absorbing system, showing only a very minor transmissive “fault” at approx. 475 nm. Even so, the authors achieved an extremely high average Vis-range (in this case 400–700 nm was chosen) optical contrast value of 79%, at a relatively high operating voltage of 4 V. Similarly, although only average coloration efficiency was achieved, attributed by the authors primarily to the poor efficiency of hole transport across the ITO layer, the device was very stable, showing only a minor loss of absorbance after more than 6000 s of repeated colouration switching.

**Scheme 1:**
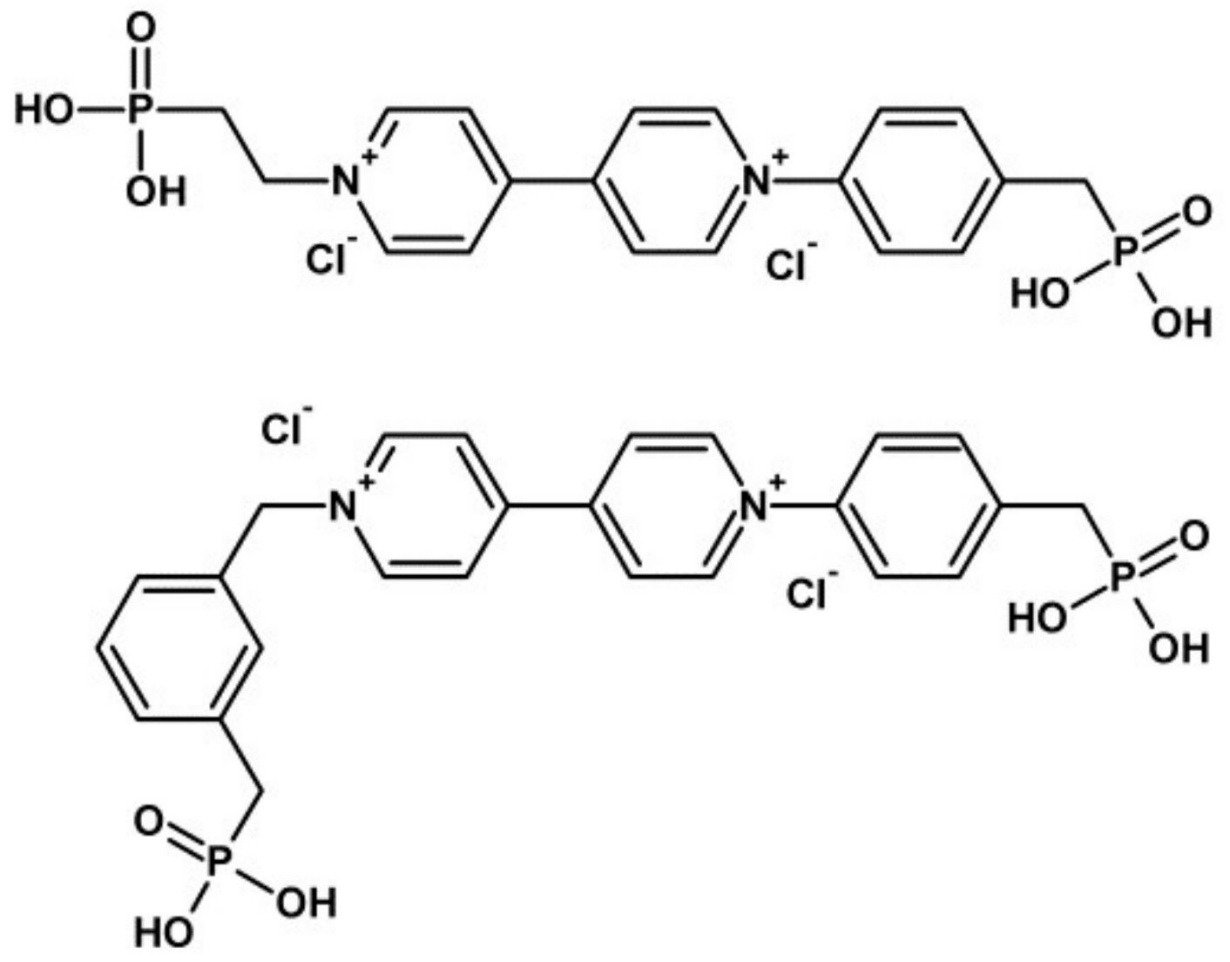
Viologen derivative electrochromes investigated by Alesanco et al. in [11].

Alesanco et al. [11] report another approach to the use of viologen derivatives as electrochromic systems (Scheme 1). In this case, the Authors focused on asymmetric derivatives, equipped with terminal phosphate groups to promote adherence to the TiO_2_ monolayer, which the authors deposited on the working electrode of their devices. Although the investigated viologen derivatives showed relatively uniform transmittance in their undoped state (above 60% across the entire Vis range), making for a good transmissive state, their reduced, coloured states in every case showed significant transmittance at approx. 500 nm and above 650 nm, making the “black” state exhibit a slight coloured hue. Both a good maximum optical contrast value of over 60% at 560 nm and excellent stability, in excess of 8000 cycles are reported. Similarly, good coloration efficiency is seen, on the order of 200 cm^2^/C. Conversely, the devices show moderately long switching times of 5–10 s, with values on the order of 30 s being implied by the presented data plots.

Rather than employing thin solid films as the active, electrochromic layers, Wang et al. [40] opted to introduce the electrochromic agents, namely a mixture of ethyl-viologen dibromide and potassium ferrocyanide, into a deep eutectic solvent gel electrolyte. The electrochromic system was investigated in the form of a gel electrolyte layer sandwiched between two Au-modified FTO electrodes. In its bleached, electroneutral state, the setup shows some absorption around 400 nm, making for a visible, yellow tint. Upon applying negative potentials (−0.9 V), a broad absorption band develops in the 500–600 nm range; it does not, however, encompass the entire visible range, showing relatively high transmittance around 450 nm and above 650 nm. Due to these features, the “dark” coloured state is more of a very dark blue than a “true black” colour. Although the authors report optical contrast values for wavelengths where it was the largest, rather than on a wavelength-average (in the Vis range) basis, an impressive value of 75% optical contrast is seen. Remarkably, after 820 potential switching cycles, carried out over the span of roughly 48 hours, this value is diminished only to approx. 60% optical contrast, evidencing moderate stability of the system. The authors also benchmarked the coloration and bleaching efficiency of their devices. The modest results obtained, establish low threshold reference points for this new electrolyte system, helping to evaluate its device performance, and prompting considerations for further application-oriented work using it.

**Scheme 2:**
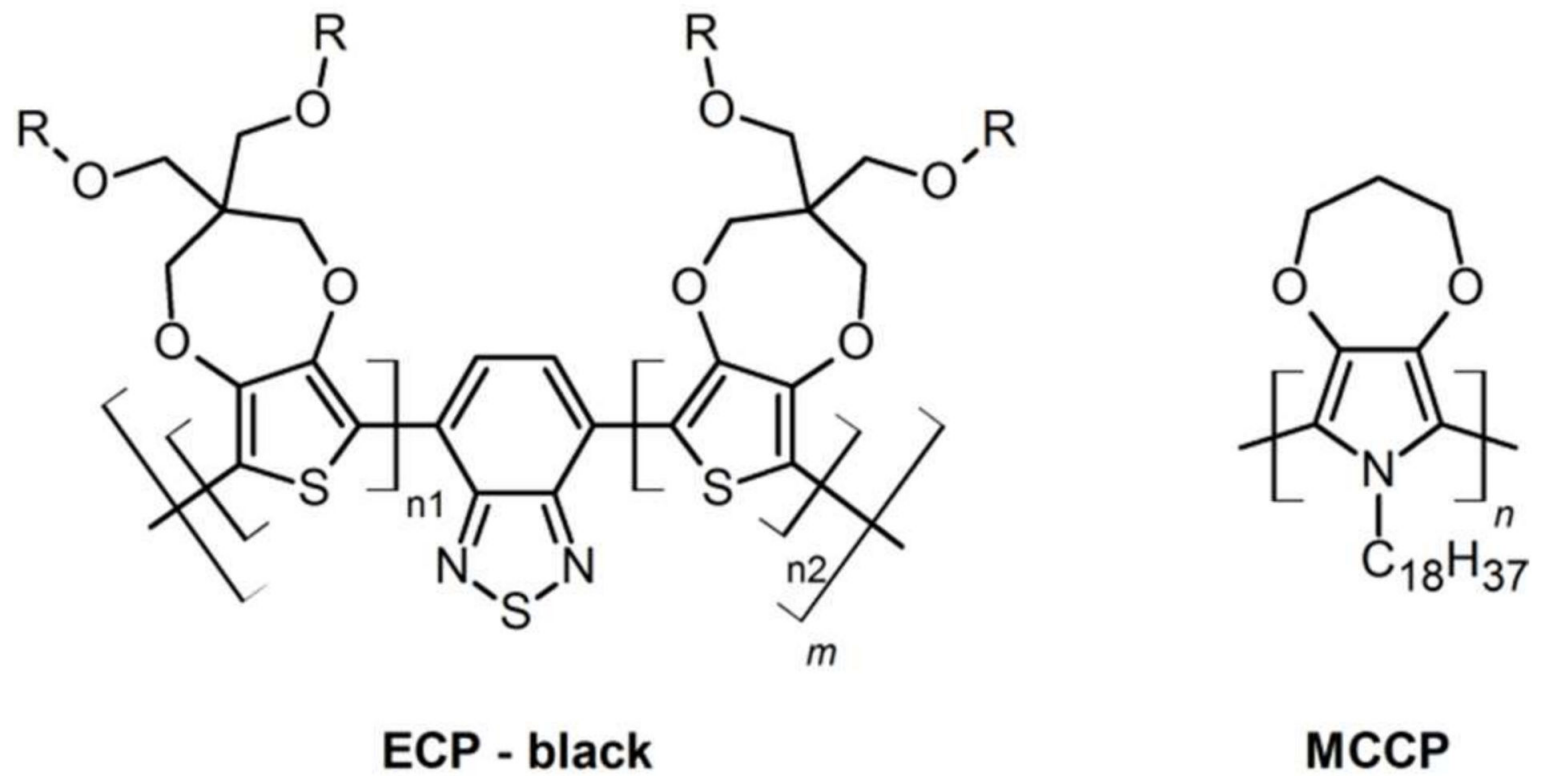
PrODOT/benzothiadiazole copolymer (ECP-Black) used as an active layer and 3,4-propylenedioxypyrrole (MCCP) used as a charge storage layer by Hassab et al. in [41].

Hassab et al. [41] report a novel approach to the architecture of electrochromic devices, while maintaining a rather conservative choice of active substances–a PrODOT/benzothiadiazole copolymer (ECP-Black) active layer and a 3,4-propylenedioxypyrrole (MCCP) (Scheme 2) supporting, charge storage layer.

By varying the thicknesses of the two layers, the authors altered their redox capacity ratio and investigated the effect of these alterations on the performance of their electrochromic devices. The consequence of such a redox capacity imbalance is that the layer with the greater capacity will be charged to a lower extent. With a sufficiently large redox capacity disparity, the layer with the greater capacity would be charged only to a negligible extent, resulting in no observable chromic changes on its part. Conversely, the layer, whose redox capacity was significantly lower, would be fully charged/discharged in the course of the operation of such a device. Interestingly, while the authors were able to achieve a significant reduction in operating voltage, the imbalanced devices achieved significantly lower optical contrast values, with 47% contrast being reported for a 1:1 layer redox capacity ratio and a rapid decrease being seen upon deviating from this ratio. Although ratios both larger and lower than 1:1 produced inferior contrasts, the effect was produced due to different considerations: if the redox capacity of the active layer was larger than that of the charge storage layer, the active layer would not be fully doped/undoped, achieving only a partially transmissive state rather than a fully transmissive one; if, on the other hand, its capacity was smaller, the thickness of the charge storage layer would contribute its own absorption, impairing the attainable transmittance of the device. A noteworthy finding was made when the authors investigated the stability of the devices and found the individual polymer films to be more stable than the device stability would imply. Investigating the redox response of the devices, the authors found that upon repeated potential switching, the potential window of each film would change and following that, the potential recorded by the reference electrode would start drifting, affecting the attainable optical contrast over time by incomplete doping/dedoping rather than polymer electro-deactivation as was the previous hypothesis. As such, while the concept of producing electrochromic devices with an unbalanced configuration shows some promise, this subject requires further investigation and development.

An interesting recent work focuses on electrochemical copolymerisation of EDOT with either of two conjugation restrained, star-shaped triazine-core, carbazole-arm derivatives [42]. Tuning the monomer feed ratio to optimise colouration efficiency, and tailoring the thickness of electrochromic layers, through careful electropolymerisation conduct, to maximise contrast ratios, afforded multielectrochromic layers, switching from clear to black through at least three intermediate hues. Although the two copolymers produced were not investigated in terms of their actual structure and composition, which would discriminate between the copolymer, or homopolymer blend mode of colouring operation, relatively good optical contrasts (up to 59% at 665–668 nm) of the electrochromic electrode deposits were achieved.

**Scheme 3:**
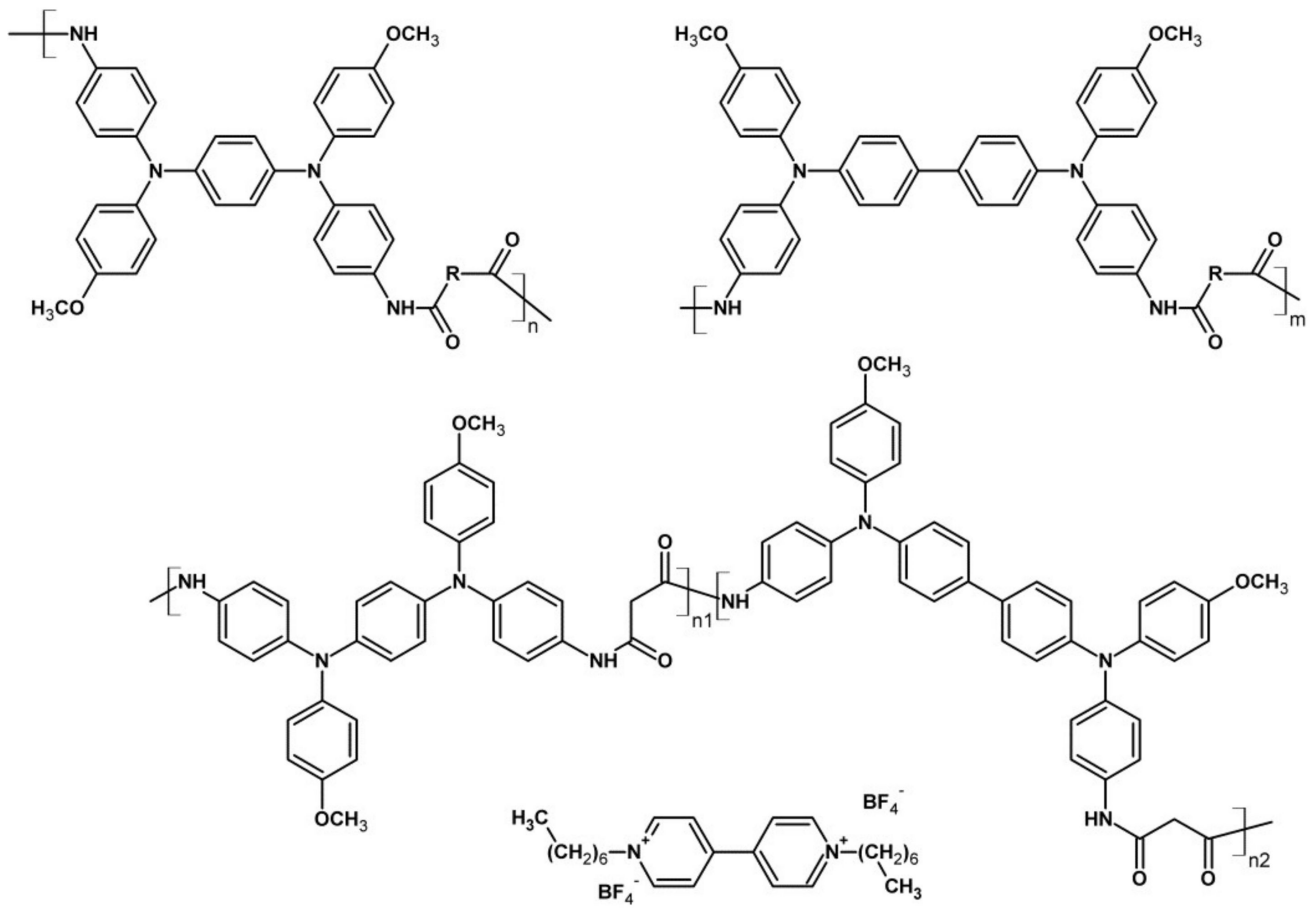
Triphenylamine derivative-based polyamides and a gel electrolyte containing an electrochromic viologen derivative, proposed by Liu et al. in [43].

A novel architecture of an electrochromic device, involving an active layer of two triphenylamine derivative-based polyamides and a gel electrolyte containing an electrochromic viologen derivative, was proposed by Liu et al. [43] (Scheme 3). The three materials were chosen based on the fact that each exhibits one of the fundamental RGB colours, with their combination being able to provide relatively uniform absorption across the entire Vis range. Interestingly, while the combination made for a deep, “true black” coloured state, it also prevented achieving a highly transmissive state, with transmittance being at most 70%, limiting the average Vis-range optical contrast to 60%. While the authors report relatively good coloration efficiency values, on the order of 170–180 cm^2^/C, the stability of the device is not particularly remarkable as roughly a 6% loss in optical density is reported after 100 potential switching cycles.

**Scheme 4:**
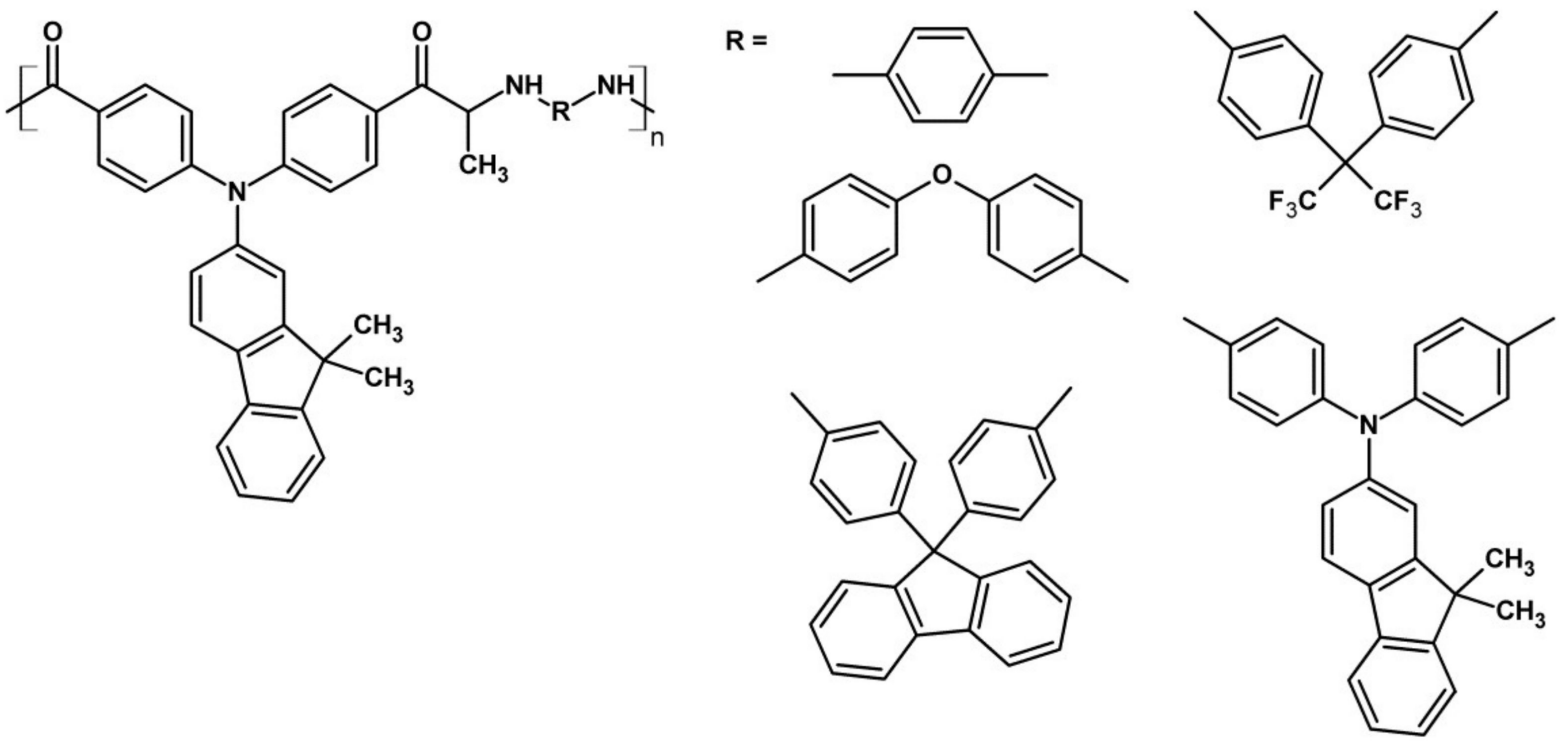
Alternant conjugated/non-conjugated polyamide copolymers, containing a novel fluorenyldiphenylamine system reported in [44].

Sun et al. [44] report interesting electrochromic devices, utilising alternating conjugated-non-conjugated polyamides, containing a novel fluorenyldiphenylamine system (Scheme 4).

The reported polyamides are readily soluble in a number of polar organic solvents, making for facile processing from solution. The compounds show only trace absorption signals above 400 nm in their uncharged state. Although doping produces noticeable Vis-range absorption, in only one case a near-black coloured state is observed, with the absorption spectrum showing distinct signals rather than broad bands.

**Scheme 5:**
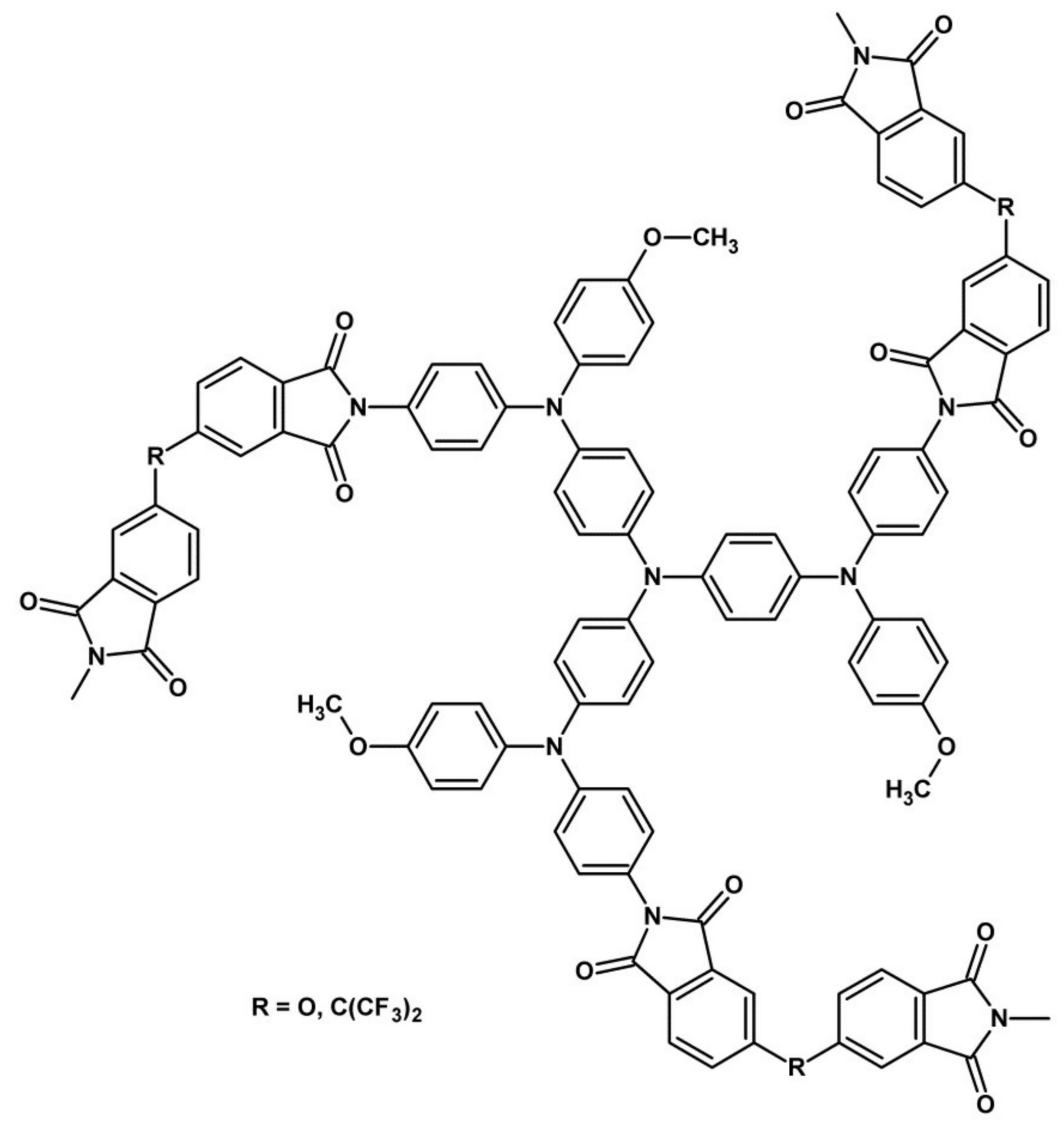
Diimide and triphenylamine moieties investigated by Sun et al. in [45].

Triphenylamine moieties, this time in conjunction with diimides rather than amides, have been used by Sun et al. [45] (Scheme 5) as components of a hyper-branched polymeric system targeted for electrochromic applications.

Interestingly, despite its high molecular weight, the polymer was found to be soluble in a number of polar organic solvents, facilitating processing and enabling the fabrication of films from solution. Although the polymer, in its electroneutral state, shows some absorption in the 300–450 nm (maximum at approx. 340 nm) range, it is low enough to make the polymer film appear transparent. Upon oxidation, the polymer adopts several transitional coloured states: yellow, red and violet, before attaining a deep black colouration at a relatively low potential (+1.30 V versus Ag/AgCl). Interestingly, the UV-Vis-NIR spectrum of that state shows almost uniform absorbance across the entire visible (400–800 nm) range, yielding a “true black” colour. Although the authors report optical contrast values for several wavelengths, no mention of the average optical contrast in the visible range is made. Another possible issue with this system is that when the potential, at which the black coloured state is achieved, the polymer layer undergoes rapid deactivation, with the observed contrast deteriorating by roughly 30% over the course of 600 s, whereas at lower potentials, even 2000 s were insufficient to produce a noticeable loss of attainable optical contrast.

Exploiting bond-coupled electron transfer (BCET) phenomenon in electrochromic devices is a novel approach, reported by Wang et al. [46], capitalising on remarkably low activation energy of this redox process.

**Scheme 6:**
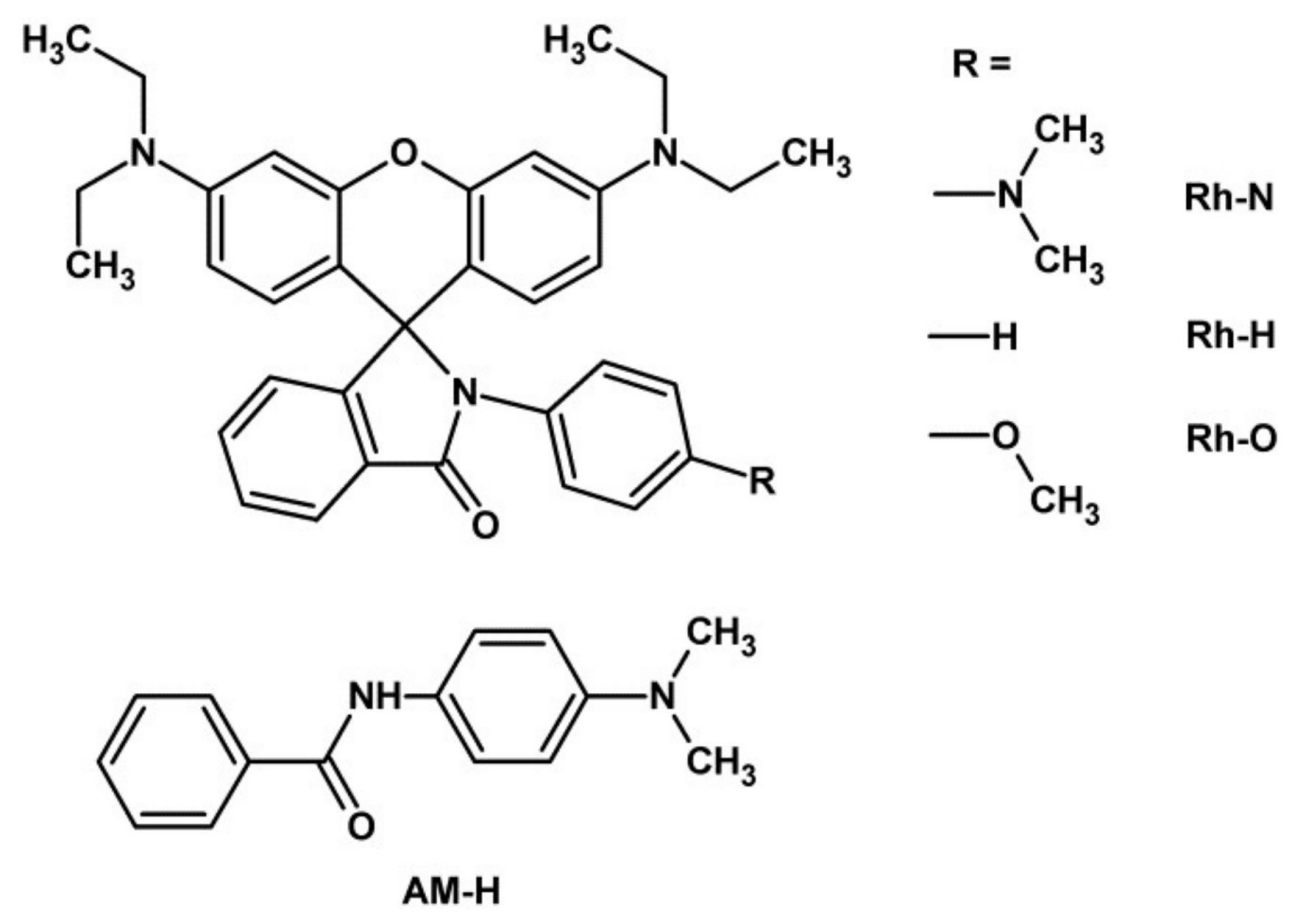
Phenylenediamine-based rhodamine systems used by Wang [46] to prospect bond-coupled electron transfer reaction in an electrochromic device.

Utilising such a reversible transition taking place in derivative of a well-known dye: rhodamine B (Scheme 6), the authors were able to produce electrochromic devices exhibiting not only extremely high coloration efficiency values, on the order of 720 cm^2^/C, but also extremely fast colour switching times, below 100 ms. The molecule capable of this reversible BCET behaviour does not contain a long conjugated bond system and shows virtually no Vis-range absorption in its electroneutral state. Although, due to this fact, the devices do not show only limited optical contrast, it is worth noting that the achieved optical contrast value is stable, even after approx. 8000 s of potential switching, a testament to the excellent reversibility of the BCET in this molecule. The device also shows a relatively high colouration efficiency of 720 cm^2^/C. As such, the use of this mechanism for systems equipped with a longer conjugated system may prove to be an original step in the development of efficient transmissive-to-black electrochromic devices.

An entirely different approach to transmissive-to-black electrochromic systems is reported by Barile et al. [47] who utilised reversible metal electro-precipitation/electro-dissolution to achieve an extremely robust device, capable of undergoing 5000 potential switching cycles (400 h under experimental conditions) with only very minor optical contrast loss. Although the authors tested different combinations of metals present in the gel electrolyte, such as Cu/Pb, and both unmodified and Pt-modified ITO electrodes, the best performance, yielding roughly 60% optical contrast and the abovementioned excellent stability, was achieved for a Cu/Ag gel electrolyte and Pt-modified ITO electrodes. Favourably, the authors have both investigated their electrochromic devices in detail and compared them to existing, commercially-available systems, achieving at least comparable parameters: a colouration efficiency of 90 cm^2^/C and a “true black” coloured state are particularly important highlights of the reported devices. Even further, the authors estimate the rare metal cost of their windows (below $0.2 and below $0.03 worth of Pt and Ag respectively are needed to produce a 1 square foot window), showing that their devices would not be a particularly cost- and resource-intensive solution.

Bearing in mind all the works and achievements discussed above, it is also necessary to take the environment, in which they will be applied, into consideration. Dussault and Gosselin’s [7] report is an example of a detailed numerical study of such considerations, as it touches on the practical aspects of utilising electrochromic windows in office spaces. An important find is that the use of electrochromic windows for north-oriented facades gives relatively little energy savings, while the savings for all other facade orientations are significantly larger and surprisingly similar to each other. Interestingly, the reasoning underlying these conclusions is a reduction in the peak load associated with cooling/air conditioning. A particularly important conclusion of the authors, after having simulated some seven thousands scenarios, is that with the use of electrochromic windows the largest energy savings can be made in warmer climates, where direct (as opposed to cloud scattered) and high solar radiation exposures are prevalent. This provides an interesting challenge and niche for developing electrochromic windows tailored for application in particular warm climate regions, e.g., tropics (devices suited to operating in high humidity) and desert/sub-desert regions (devices suited to operating in extremely low humidity and resistant to large day/night temperature variations).

## 3. Summary and Conclusions

Electrochromism of conjugated polymers has been discovered alongside the development of electrochemical methods for the preparation of conductive polymers and results from changes of the electronic structure of the polymer during its redox doping. Initially, high hopes were had for swift application of these materials in electrochromic displays. This appears to still be feasible, but the nature and properties of most polymer-based transmissive to black ECDs makes them more suited to application in either static displays or “smart windows”, the latter being particularly promising.

The ability to produce layers exhibiting transparent to black electrochromism is highly sought after and numerous solutions have been proposed and reported. Most commonly, they rely on mixing several polymers into a composite material or on the preparation of copolymers. The methods and individual efforts (focusing on the most recent progress) to design the best transmissive to black device solution have been described in this work, each bearing its own advantages and shortcomings.

The most straightforward solution is to select such components of a composite material or copolymer that their absorbance in the doped (or undoped) state will cover the whole visible light spectrum. This approach, however, is rather demanding as it is difficult to find components, which will be transparent in their undoped (or doped) state. Furthermore, the use of numerous constituents may lead to issues of their differing chemical and thermal stability, possibly, convoluting the manufacturing of such devices.

The most effective solution to date is to prepare a polymer from a specifically designed monomer. In this case, synthetic ability is the limiting factor, as the monomer must fulfil a number of conditions for successful application in a transparent to black electrochromic device. The monomer must polymerise readily, yield a soluble (processable) and resilient polymer, which should undergo colour change rapidly as a result of mild electrical stimulation.

Improving upon the above, the prudent approach appears to be to design and prepare a macromonomer containing functional groups tailored towards providing the desired Visible-range colouration. This approach allows control over the structure of the resultant copolymer, alleviating some of the issues described for copolymers. The approach enables preparation of multi-coloured electrochromic materials, transparent to black systems among them.

Currently, the number of systems strictly adhering to the above requirements is limited. Nevertheless, new systems are being developed and investigated, potentially fulfilling the criteria set for a transparent-to-black electrochromic device. The key to this challenge lies in unlocking the relationships between the molecular and electronic structure of π-conjugated scaffolds at their discrete redox states. A means to this end will involve interdisciplinary research efforts into bespoke molecular targets across materials science, chemistry and physics disciplines. As more and more knowledge puzzle pieces find their right match in the grand picture of organic electrochromism, procurement of the much anticipated materials is but a matter of time.
